# New Appliances and Things Medical

**Published:** 1905-08-12

**Authors:** 


### NEW APPLIANCES AND THINGS MEDICAL.

### LOZAL HOSPITAL SHEETING.

(Burgoyne, Burbidges and Company, London.)

The superiority of rubber sheeting for surgical and nursing purposes, over the various substitutes which have been put forward from time to time, is scarcely open to question. Nevertheless, it must be admitted that the common kinds of mackintosh sheeting have the disadvantage that they deteriorate when subjected to vigorous attempts at disinfection. This drawback has now been minimised by Messrs. Burgoyne, Burbidges and Company, who by improved processes have been able to manufacture a sheeting which is very durable, is undamaged by strong antiseptics, and which can even be boiled in water for a short period without being destroyed. Lozal, which is the name they have given to this material, is made single-proofed and double-proofed. The latter, a sample of which has been submitted to us, is a good heavy sheeting, which shows no tendency to form creases, and which admirably serves the purposes for which it is intended. We especially commend Lozal sheeting for use in hospitals, where durability is so necessary to good economy.

### SANATOGEN.

(The Sanatogen Co., 83 Upper Thames Street, London, E.C.)

The preparation before us is a compound of casein, the essential proteid constituent of milk with glycero phosphoric acid, and may chemically be regarded as sodium-casein-glycero-phosphate. It contains, according to König, 88·63 per cent. of proteids, of which 80·9 are soluble. The feature, however, which distinguishes it from all other proteid preparations on the market is the quantity of organic phosphorus which it contains; this amounts to 1·32 per cent. (P.). According to Lehmann and Hempel, cow's casein contains ·84 per cent. of phosphorus, which would mean that of the organic phosphorus contained in Sanatogen about half is present as a constituent of the casein and about half in the form of glycero-phosphate. The therapeutic rôle of organic phosphorus is at present attracting much attention, more especially the behaviour of this variety of phosphorus as compared with that of inorganic phosphorus or the ordinary alkaline or alkaline earth phosphates, such for instance as the official calcium phosphate ($Ca_3(PO_4)_2$). The balance of evidence at present seems to be that the inorganic phosphates are not capable of supplying the phosphorus needs of the organism, the body being unable to build up from this simpler form of phosphorus the complex nucleins and phosphorised fats which are essential for the functionings of the nervous system. Sanatogen has received an extensive trial at the hands of the profession, and it is quite evident from the literature that the proteid and the phosphorus contained in this substance are capable of producing both a nutritive and therapeutic effect. In the recent typhoid epidemic at Lincoln it was found (*Lancet*, June 1905) that sanatogen could be given with advantage to patients still the subjects of slight pyrexia, and certainly exerted a beneficial influence upon their nutrition. These results at Lincoln confirm the previous work of Professor Ewald. We have no hesitation in saying that sanatogen is worthy of, and likely to take, a very prominent place in invalid dietetics. We have entered at some length into the question of its phosphorus, because we believe that this element exerts an important influence in its action.

